# Biosynthesis of Rhamnosylated Anthraquinones in *Escherichia coli*

**DOI:** 10.4014/jmb.1911.11047

**Published:** 2019-12-24

**Authors:** Trang Thi Huyen Nguyen, Hee Jeong Shin, Ramesh Prasad Pandey, Hye Jin Jung, Kwangkyoung Liou, Jae Kyung Sohng

**Affiliations:** 1 Department of Life Science and Biochemical Engineering, Sun Moon University, Asan 3460, Republic of Korea; 2 Department of Pharmaceutical Engineering and Biotechnology, Sun Moon University, Asan 31460, Republic of Korea

**Keywords:** Rhamnosyltransferase, quinizarin, *Saccharothrix espanaensis*

## Abstract

Rhamnose is a naturally occurring deoxysugar present as a glycogenic component of plant and microbial natural products. A recombinant mutant *Escherichia coli* strain was developed by overexpressing genes involved in the TDP-_L_-rhamnose biosynthesis pathway of different bacterial strains and *Saccharothrix espanaensis* rhamnosyl transferase to conjugate intrinsic cytosolic TDP-_L_-rhamnose with anthraquinones supplemented exogenously. Among the five anthraquinones (alizarin, emodin, chrysazin, anthrarufin, and quinizarin) tested, quinizarin was biotransformed into a rhamoside derivative with the highest conversion ratio by whole cells of engineered *E. coli*. The quinizarin glycoside was identified by various chromatographic and spectroscopic analyses. The anti-proliferative property of the newly synthesized rhamnoside, quinizarin-4-O-α-_L_-rhamnoside, was assayed in various cancer cells.

Anthraquinones are naturally occurring aromatic organic compounds found in plants, fungi and actinomycetes [1]. The dihydroxyanthraquinones constitute the most important group and are largely used as dyes and in the manufacture of dye intermediates [2, 3]. In addition, anthraquinone derivatives exert a wide range of biological activities [4-10]. They have also been used as anticancer agents to treat breast cancer and acute leukemia [11, 12].

Emodin (1,3,8-trihydroxyl-6-methylanthraquinone), which is an active compound isolated from several Chinese herbs, is traditionally used as a laxative agent. Treatment with emodin has been shown to result in body weight reduction, lipid-lowering, blood glucose control, and anti-inflammatory effects [13]. Danthron (1,8-dihydroxyanthraquinone) was isolated from the root and the rhizome of *Rheumpalmatum L.*, used in traditional medicine [14]. Alizarin red S is used for histological characterization of calcium deposits [15]. Quinizarin occurs as a glycoside in small amounts in the root of the madder plant, *Rubia tinctorum* [16], and is used as a fungicide and pesticide [17] as well as an inhibitor of tumor cell growth [18]. It is an inexpensive dye used to color gasoline and heating oil; it also acts as an intermediate for the synthesis of indanthrene- and alizarin-derived dyes [2]. Further, anthraquinone glycosides exhibit stronger activity than free aglycones [19].

*Saccharothrix*, a member of the order *Actinomycetales* [20], generates glycosylated natural products. The *S. espanaensis* genome carries 106 glycosyltransferase (GT) genes [21]. One of the GTs has been recently characterized as a promiscuous rhamnosyl transferase (7665) [21], which glycosylates anthraquinones using thymidine diphosphate (TDP)-L-rhamnose as a sugar donor. With the aim of producing different rhamnoside derivatives of anthra-quinone, we used an *Escherichia coli* BL21 (DE3) Δ*pgi*Δ*zwf*Δ*galU* strain [22] developed by blocking glucose phosphate isomerase (*pgi*), glucose-6-phosphate dehydrogenase (*zwf*), and uridylyltransferase (*galU*) genes to divert carbon flow from glucose to TDP-_L_-rhamnose via G-1-P and dTDP-glucose. *E. coli* BL21 (DE3) Δ*pgi*Δ*zwf*Δ*galU* was further engineered by introducing TDP-_L_-rhamnose biosynthesizing genes harboring plasmids pCDFDuet-TGSDH and pACYCDuet-EPKR, carrying genes for biosynthesis of TDP-_L_-rhamnose (*tgs*: TDP-glucose synthase from *Thermus caldophilus* GK24; *dh*: dTDP-D-glucose 4,6-dehydratase from *Salmonella typhimurium* LT2; *epi*: TDP-4-keto-6-deoxyglucose 3, 5-epimerase from *Streptomyces antibioticus* Tü99; and *kr*: TDP-glucose 4-ketoreductase from *S. antibioticus* Tü99) [23, 24] and pET28a (+)-7665 carrying a rhamnosyltransferase from *S. espanaensis* (7665) [21] (Fig. S1). The fully grown cells of engineered *E. coli* were used to biotransform five anthraquionones into their respective rhamnosides (Fig. 1). The anti-proliferative activity of quinizarin rhamnoside was assessed and the results were significant compared with those of the corresponding aglycone.

First, we employed a versatile post-biosynthesis modifying enzyme, O-rhamnosyltransferase (7665), derived from *S. espanaensis* for the biosynthesis of anthraquinone rhamnosides in the engineered *E. coli* mutant strain overexpressing genes for TDP-_L_-rhamnose. Five different anthraquinones were added for biotransformation into respective O-rhamnosides. After 20 h of isopropyl β-D-1-thiogalactopyranoside (IPTG)-induced culture, we supplemented anthraquinone exogenously at a final concentration of 0.2 mM. The biotransformation reaction was continued for the next 28 h at 20°C, followed by extraction using a double volume of ethyl acetate and analysis via high-performance liquid chromatography (HPLC-PDA).

The HPLC-PDA analysis of each sample yielded product peaks at shorter retention times (*t*_R_) than the substrate peak in each reaction mixture, as expected. New peaks appearing at *t*_R_ ~ 19.6 min in alizarin (*t*_R_ 23.3 min); *t*_R_ ~ 21.06 min in anthrarufin (*t*_R_ 27.59 min); *t*_R_~ 21.24 min in chrysazin (*t*_R_ 26.77 min); *t*_R_ ~ 23.53 min in emodin (*t*_R_ 25.9 min); and *t*_R_~ 21.2 min in quinizarin (*t*_R_ 27.4 min) at the UV absorbance of 420 nm were suspected to be rhamnose-conjugated derivatives (Fig. 2). These samples were further analyzed by high-resolution quadruple time-of-flight electrospray ionization (HR-QTOF ESI/MS) to confirm the conjugation of rhamnose moiety with each anthraquinone substrate added exogenously. The mass spectra displayed an exact mass of emodin *m/z* 271.06 [M+H]^+^, while the mass spectrum of *m/z* 417.11 [M+H]^+^ resembled the rhamnose-conjugated derivative of emodin. Similarly, chrysazin, quinizarin, anthrarufin and alizarin conjugated to rhamnose *m/z* 409.08 [M+H]^+^ were established based on the mass analysis of respective product peaks. The mass spectra were obtained along with their sister fragments of chrysazin, quinizarin, anthrarufin and alizarin *m/z* 241.05 [M+H]^+^ (Fig. S2). The biotransformation reaction analysis by HPLC-PDA and ESI/MS revealed that the engineered strain converted all exogenously supplemented substrates to products. The conversion percentage of emodin, chrysazin, quinizarin, anthrarufin and alizarin were 2.4%, 2.5%, 17%, 10.7%, and 3%, respectively. Based on the highest conversion, a further study of quinizarin alone was carried out.

We increased the bioconversion of quinizarin via supplementation of different concentrations (0%, 2%, 4%, 6%, and 8%) of glucose in cultures grown under identical conditions during biotransformation. The change in conversion percentage of quinizarin to product was monitored at different time intervals (from 0 to 60 h). The result showed that supplementation of 2% additional glucose improved the conversion from 22% (36 h, without additional glucose) to 75% (48 h) (Figs. S3 and S4). The addition of glucose facilitated cell growth and product yield.

The product was purified by using prep-HPLC and then subjected to nuclear magnetic resonance (NMR) analyses. While comparing the ^1^H NMR spectra of standard quinizarin and the reaction product, signals from the parent compound containing 2-hydroxyl groups in the symmetrical position were detected at δ 12.71 (1H, *s*) while in the reaction compound hydroxyl group, the signals were detected at δ 12.88 (1H, s) (Table 1, Figs. S5a and S5b). The anomeric proton (1’-H) was consistent with δ 5.48 (*d, J* = 1.7 Hz, 1H); however, the anomeric proton coupling constant (*J* = 1.7 Hz) confirmed that the conjugation of rhamnose moiety was in α-configuration. In addition, based on the ^13^C NMR analysis of the reaction product, the new peak appeared at δ 100.01 ppm for anomeric carbon and other carbon peaks between 70 and 80 ppm along with a CH_3_ peak at 18.3 ppm. All the peaks were assigned to their respective carbon as shown in Figs. S6a and S6b. To confirm the position of sugar conjugation, we analyzed ^1^H-^13^C correlation using heteronuclear single quantum coherence (HSQC) and heteronuclear multiple bond correlation (HMBC) spectroscopy. The result supported the correlation between the observed anomeric carbon and anomeric proton revealed by HSQC (Fig. S7). Similarly, the carbon C-4 of the quinizarin signal appearing at δ 150.48 ppm showed a direct correlation with the observed anomeric proton at δ 5.48 ppm in HMBC (Fig. S8). Based on these results, the product was established as quinizarin 4-O-α-_L_-rhamnoside.

Previous studies showed that the anticancer effects of anthraquinones were associated with the suppression of cancer cell proliferation [25]. We thus evaluated the effects of quinizarin and its derivative on the proliferation of A375SM melanoma, AGS gastric cancer, MCF-7 breast cancer, and U87MG brain cancer. The inhibitory effect of quinizarin rhamnoside was greater than that of aglycone in all cancer cell lines tested. This result showed that approximately 70% of AGS gastric cancer cells failed to grow in the presence of 50 μM concentration of quinizarin rhamnoside while the suppression of cell growth was only 20% under the same concentration of quinizarin. Although subtle growth reduction was observed with rhamnoside derivative, the decrease in cell proliferation of MCF-7 breast cancer cells and U87MG brain cancer was not significantly different with quinizarin and its rhamnoside derivative (Fig. 3).

Chemical synthesis of anthraquinone glycosides requires multiple steps, uses hazardous chemicals, and is therefore an environmentally unfriendly approach [26]. Moreover, production of anthraquinone rhamnosides in practical quantities from plant sources has been tedious and impractical as biosynthesis in large quantity from these sources is difficult to achieve while purification and extraction are more challenging because of the presence of a large number of other metabolites [27]. Therefore, regiospecific biosynthesis using engineered recombinant microbial cells is superior in terms of sustainability while being eco-friendly and enabling easy fermentation and scale-up for industrial biosynthesis [28]. Thus, this study provides a broad overview of the modification of anthra-quinones by rhamnosylation using an engineered *E. coli* strain in a sustainable way. The antiproliferative activities of the newly synthesized molecule prompted further investigations into the search for novel molecules in medicinal chemistry.

## Supplemental Materials



Supplementary data for this paper are available on-line only at http://jmb.or.kr.

## Figures and Tables

**Fig. 1:**
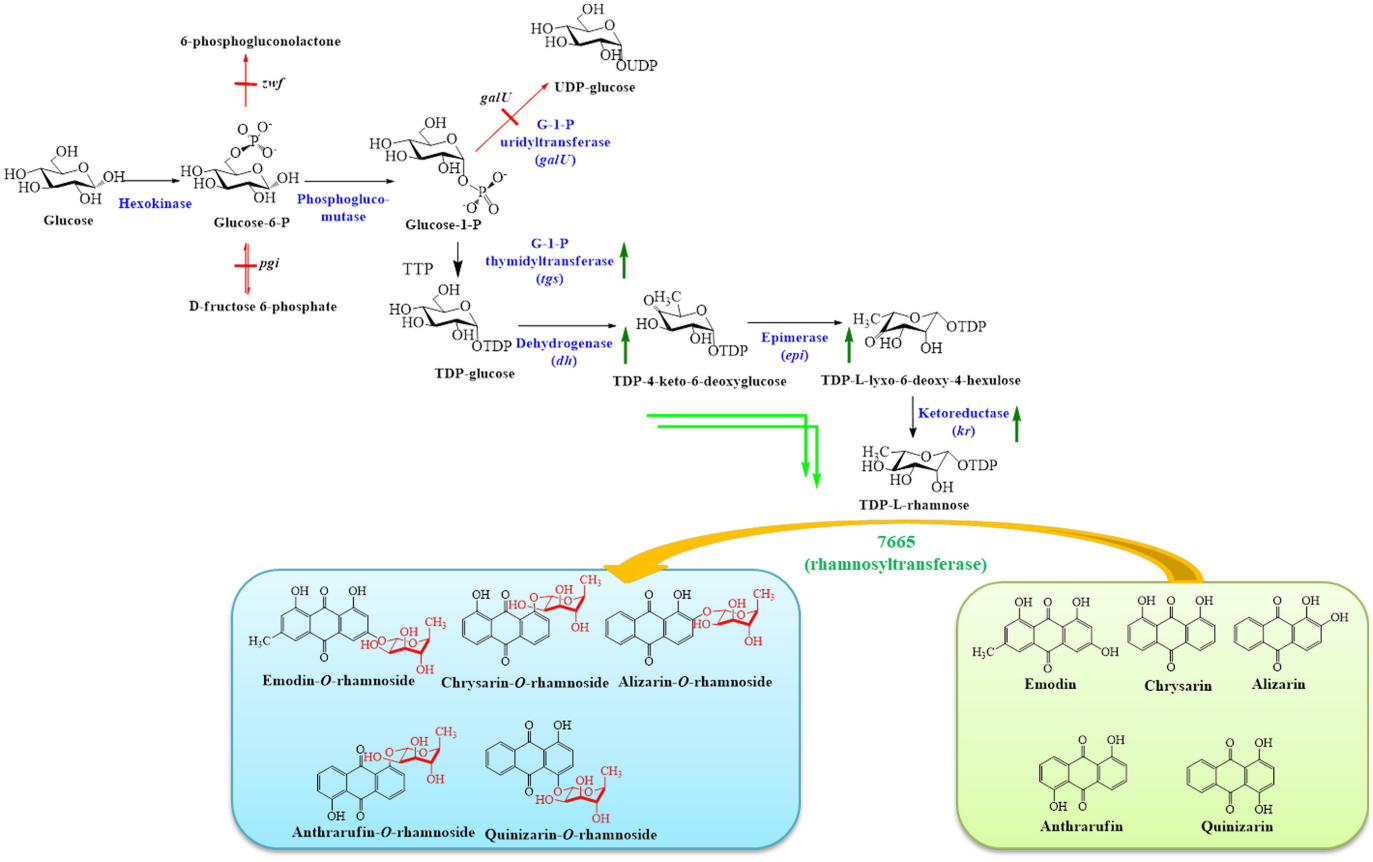
Schematic diagram representing the metabolic engineering of *E. coli* BL21(DE3) for the biosynthesis of rhamnoseconjugated anthraquinones. The genes (*pgi*, *zwf*, and *galU*) were knocked out of the genome. The dTDP-_L_-rhamnose was generated in the cytosol of engineered *E. coli* by overexpressing the respective genes in the sugar pathway. Rhamnosyl transferase (7665) from *S. espanaensis* was used for the conjugation of sugar to the exogenously supplemented anthraquinones (emodin, chrysazin, alizarin, anthrarufin, and quinizarin).

**Fig. 2:**
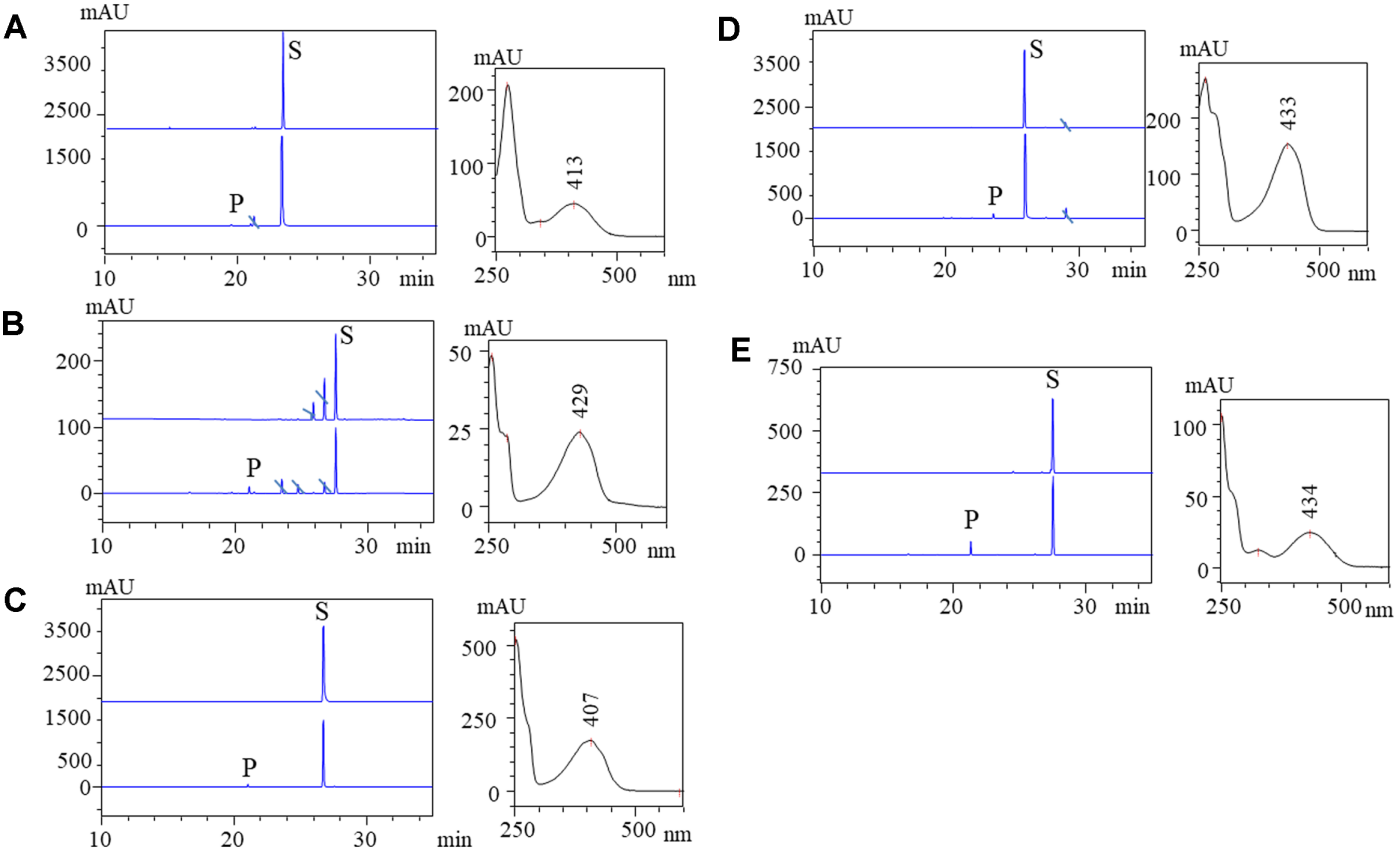
HPLC-PDA chromatogram of biotransformed reaction mixtures compared with respective standards. S refers to the substrate peak and P refers to the product. (**A**) alizarin (**B**) anthrarufin, (**C**) chrysazin, (**D**) emodin, (**E**) quinizarin.

**Fig. 3:**
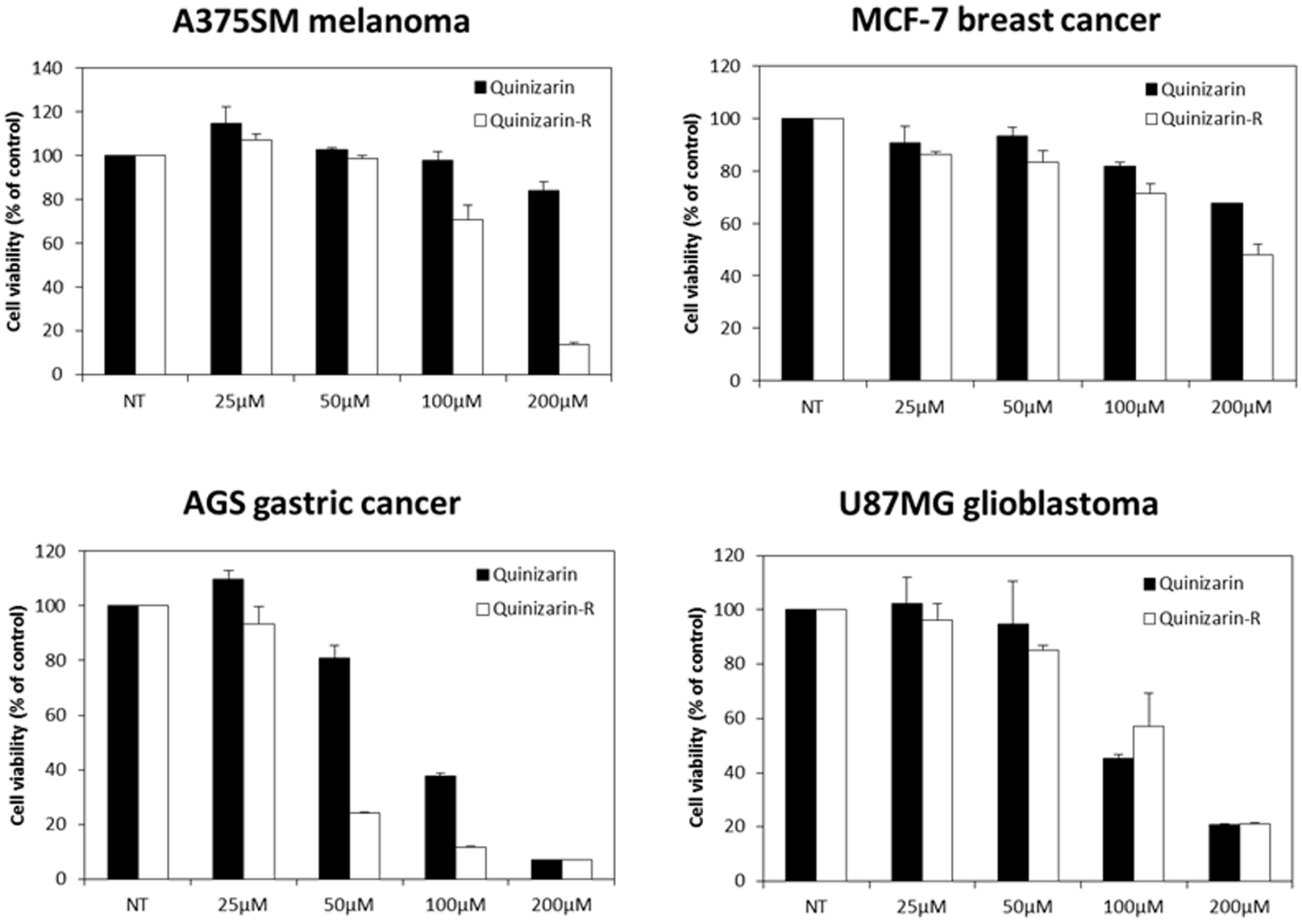
Inhibitory effects of quinizarin and quinzarin-4-*O*-α-_L_-rhamnoside derivative (denoted as quinizarin-R) on cancer cells including AGS gastric cancer, A375SM skin cancer, MCF-7 breast cancer and U87MG brain cancer.

**Table 1:** Comparison of ^1^H- and ^13^C-NMR spectra of quinizarin and quinizarin-4-*O*-α-_L_-rhamnoside measured in DMSO-*d6*.

	^1^H-NMR			^13^C-NMR

Position	Quinizarin	(quinzarin-4-*O*-*α*-L-rhamnoside)	quinizarin	(quinzarin-4-*O*-*α*-L-rhamnoside)
1-OH	12.71 (*s*,1H)	12.88 (*s*,1H)	158.57	157.89
2	7.45 (*s*,1H)	7.38 (*d*, *J*=9.3 Hz,1H)	131.24	129.48
3	7.45 (*s*,1H)	7.38 (*d*, *J*=9.3 Hz,1H)	131.24	127.20
4-OH	12.71 (*s*,1H)	-	158.57	150.48
5	7.98 (*dd*, *J* = 5.8, 3.3 Hz, 1H)	7.90 (*d*, *J* = 32.7 Hz, 1H)	128.57	126.54
6	8.27 (*dd*, *J* = 5.8, 3.3 Hz, 1H)	8.16 (*dd*, *J* = 34.9, 7.6 Hz, 2H )	134.78	134.24
7	8.27(*dd*, *J* = 5.8, 3.3 Hz, 1H)	8.16 (*dd*, *J* = 34.9, 7.6 Hz, 2H)	134.78	132.31
8	7.98 (*dd*, *J* = 5.8, 3.3 Hz, 1H)	7.90 (*d*, *J* = 32.7 Hz, 1H)	128.57	126.20
9			188.59	188.85
10			188.59	180.95
11			114.58	116.01
12			114.58	120.59
13			136.99	135.60
14			136.99	134.87
1′		5.48 (*d*, *J* = 1.7 Hz, 1H)		100.01
2′		3.96 (*dd*, *J* = 4.4 Hz, 1H)		72.82
3′		3.36 (dd, *J* = 9.1, 4.5 Hz, 1H)		75.05
4′		4.81 (*d*, *J* = 5.8 Hz, 1H)		70.59
5′		4.06 (*s*,1H)		72.17
6′-CH_3_		1.12 (*s*,1H)		18.31

Multiplicities are indicated by s (singlet), d (doublet), t (triplet), q (quartet), and m (multiplet), including coupling constant *J*.
