# Supplementary material for: Biosynthesis of Rhamnosylated Anthraquinones in Escherichia coli
Source: J Microbiol Biotechnol. 2019 Dec 24;30(3):398–403. doi: 10.4014/jmb.1911.11047 (PMC9728250; doi:10.4014/jmb.1911.11047)
Supplement: Supplementary file 1 [file JMB-30-3-398-supple.pdf]

J. Microbiol. Biotechnol.
https://doi.org/10.4014/jmb.1911.11047

jmb

Supplementary Information 19-11047

Materials and Methods

General Procedures

Anthraquinones were purchased from Tokyo Chemical Industry (Tokyo, Japan). *Escherichia coli* BL21(DE3) *ΔpgiΔzwfΔgalU* [1] was used as expression and biotransformation host. Luria-Bertani (LB) plates and broth media supplemented with appropriate antibiotic (streptomycin 50 µg/mL, chlophenicol 50 µg/mL, kanamycin 50 µg/mL) was used for the *E. coli* growth, colony selection, culture preparation, and biotransformation. Fermentation was carried out in LB medium. Previously constructed recombinant plasmid pET28a (+)-7665, donated by Prof. Andreas Bechthold, University of Freiburg, Germany [3] was used along with pCDFDuet-TGSDH and pACYCDuet-EPKR [2] were used for the recombinant strain construction. The recombinant plasmids were reconfirmed by restriction endonuclease digestion prior to use.

Culture preparation and whole cell biotransformation

Seed culture of *E. coli* strain harboring plasmids pET28a(+)-7665, pCDFDuet-TGSDH and pACYCDuet-EPKR was prepared in 5 mL LB medium supplemented with streptomycin, chloramphenicol, and kanamycin. The culture was incubated at 37 ^{o}C for 3-4 h. Approximately 500 µL of seeds was transferred to the same medium (50 mL) and incubated at 37 ^{o}C until the cells' optical density 600nm (OD_{600nm}) reached 0.5-0.7. To this growing culture, 0.5 mM isopropyl β-D-1-thiogalactopyranoside (IPTG) was added to induce protein expression, followed by incubation for 18 h at 20 ^{o}C. Anthraquinones (alizarin, emodin, chrysarin, anthrarufin, and

quinizarin) were dissolved in dimethysulfoxide (DMSO) to prepare stock concentration of 50 mM for exogenous supply. Each substrate at a concentration of 0.2 mM was fed and was allowed to biotransform into the respective products. After 48 h of biotransformation reaction, all the biotransformation cultures (including controls) were extracted by double volume of ethyl acetate in separating funnel followed by the addition of double volume of ethyl acetate and vertical shaking for 30 min. Then the aqueous and organic layers of the cultures were allowed to settle for another 30 min. The organic ethyl acetate layer was transferred and evaporated using rotatory evaporator. The final remaining sample was dissolved in 1 mL methanol. Most of the protocols followed were similar to those described in our previous reports [4]. This sample was directly analyzed using a high performance liquid chromatogram connected to a photo diode array (HPLC-PDA) and high-resolution quadruple time-of-flight electrospray ionization-mass spectrometry (HQ-QTOF ESI/MS) analysis.

Analytical procedures

From the aforementioned samples, 20 µL was injected and directly analyzed by HPLC-PDA (Shimadzu, Japan; SPD-M20A Detector) using a reverse phase C_{18} column (Mightysil- RP-18GP, 150 x4.6mm, Kanto Chemical, Japan). The binary mobile phase were composed of solvent A (HPLC grade water + 0.05% TFA) and solvent B (100% acetonitrile, ACN). Total flow rate was maintained as 1 mL/min for 35 minute program. ACN concentrations were 10% (0–10 min), 20% (10–25 min), 100% (25–28 min), 70% (28–30 min), and 10% (30–35 min).

The products were purified by prep-HPLC with a C_{18} column (YMC-Pack ADS-AQ (250× 20 mm I.D., 10 µm) connected to UV detector at 420 nm using a 35 min binary program with ACN concentrations were as follows 10% (0-10 min), 20% (10-15 min), 50% (15- 20 min), 70% (20-25 min), 90% (25-30 min), 50% (30-34 min), 20% (34-35 min). The total flow of

solvent was 10 mL/min. The purified products were then dried completely, lyophilized and were used for structural elucidation and bioactivity.

The high resolution quadruple time-of-flight electrospray ionization-mass spectrometry (HR-QTOF ESI/MS) spectra were obtained on ACQUITY (UPLC; Waters, MA, USA) coupled with SYNAPT G2-S (Waters). For structural elucidation of biotransformed metabolite, the samples including the standard were dissolved in dimethyl-sulfoxide-d_6 (Sigma-Aldrich MO, USA). The nuclear magnetic resonance (NMR) was done by analyzing their ^{1}H, ^{13}C NMR with several 2D NMR spectroscopies (heteronuclear single-quantum correlation [HSQC] and heteronuclear multiple-bond correlation [HMBC]). Standard molecule was analyzed with a 300 MHz Brucker (Germany) BioSpin NMR. The raw fid files were processed by using MestReNova 11.0 program (Mestrelab Research S.L., Santiago de Compostela, Spain).

Antiproliferative activities

AGS gastric carcinoma cells were maintained in RPMI 1640 medium containing 10% fetal bovine serum (FBS). A malignant melanoma cell line, A375SM, was obtained from the Korean Cell Line Bank (Seoul, Korea) and cultured in Dulbecco's modified Eagle's medium (DMEM; HyClone; GE Healthcare Life Sciences, Logan, UT, USA) supplemented with 10% (v/v) heat-inactivated fetal bovine serum (FBS; Rocky Mountain Biologicals, Inc., Missoula, MT, USA), 10 U/mL penicillin and 100 μg/mL streptomycin (Cellgro Mediatech; Corning Incorporated, Corning, NY, USA), and 10 mM HEPES (Invitrogen; Thermo Fisher Scientific, Inc., Waltham, MA, USA) at 37°C in 5% CO_2 and 95% air in a humidified cell incubator. Melanoma cells were trypsinized using 0.05% trypsin/0.02% EDTA [5]. MCF-7 cells were obtained from the American Type Culture Collec tion (Rockville, MD) and grown in monolayer

in Nunc culture dishes (six wells of 9.6 cm^2/plate). Cells were maintained in Dulbecco's minimum essential medium (Gibco) supplemented with 5% PCS (Gibco Bio-Cult, Paisley, Scotland), glutamine (2 mmol/liter), streptomycin (100 μg/mL) and penicillin (100 IU/mL), and phenol red as pH indicator [6]. U87MG human glioblastoma cells, were derived from a primary brain tumor of a 44 year old Caucasian woman, were obtained from American Type Culture Collection (ATCC, Manassas, VA). These adherent cells contain epithelia morphology. The cells propagated in Eagle's Minimun Essential media (EMEM) supplemented with 10% fetal bovine serum and 1% penicillin/ streptomycin (Sigma, St. Louis, MO mixture and maintained at 37 °C and 5% CO_2 [7]. All cells were maintained at 37 °C in a humidified 5% CO_2 incubator.

For cell growth assay, various cancer cells were plated at 2×10^3 cells/well in 96-well culture plates. The compounds were added to each well with various concentrations and the cells were incubated for 72 h. Cell growth was measured using a 3-(4,5-dimethylthiazol-2-yl)-2,5-diphenyltetrazolium bromide (MTT) colorimetric assay. 50 μl of MTT (2 mg/ml stock solution) was added and the plates were incubated for an additional 4 h. After removal of medium, 100 μl of DMSO was added. The absorbance was measured at 540 nm using a microplate spectrophotometer (Thermo Scientific Multiskan® Spectrum).

Fig S1. Details of recombinant plasmids used in this study.

TDP-glucose synthase (tgs) and TDP-glucose 4,6-dehydratase (dh) were cloned in pCDFDuet-1/Smr vector to form pTGSDH with the restriction enzyme BamHI/HindIII and BglII/EcoRV, respective .

TDP-4-keto-6-deoxyglucose 3,5-epimerase(epi) and TDP-glucose 4-ketoreductase (kr) were cloned in pACYC-Duet-1/Cmr vector with the restriction enzyme BamHI/HindIII and EcoRV/XhoI, respective to develop the pAC-EPKR recombinant.

The rhamnosyltransferase 7665 cloned into the NheI/HindIII digested vector pET28a(+) to complete the cassettle [2.3].

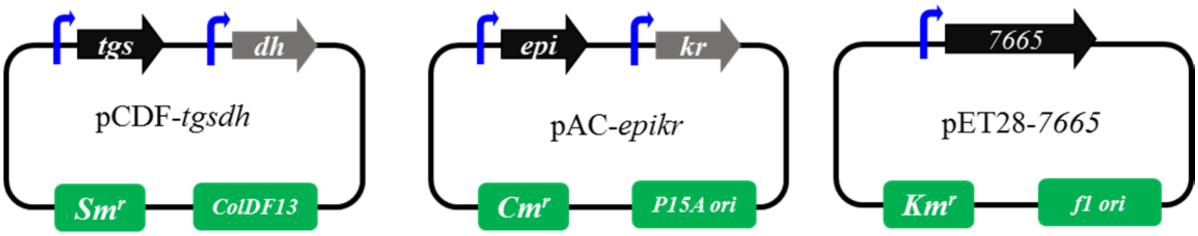

Fig S2. HR-QTOF ESI/MS analyses of glycosylated (A) alizarin (B) anthrarufin, (C) chyrasin, (D) emodin, (E) quinizarin confirmed by comparing the mass fragments.

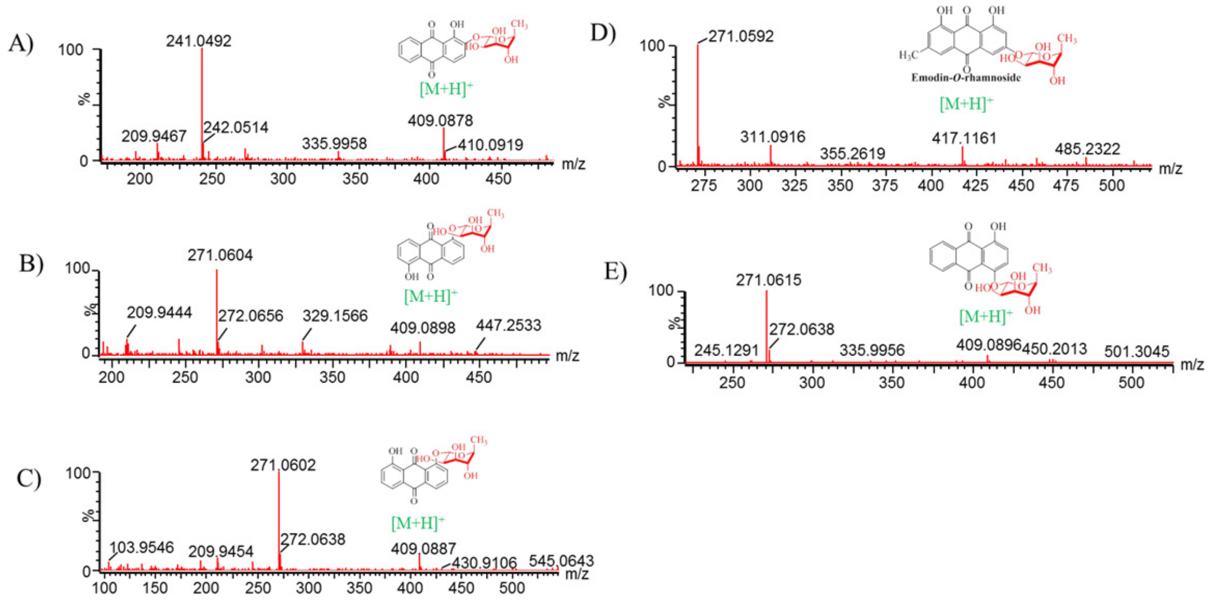

Fig S3. Production profile of quinizarin- *O*- rhamnoside at different incubation time intervals and glucose supplementations.

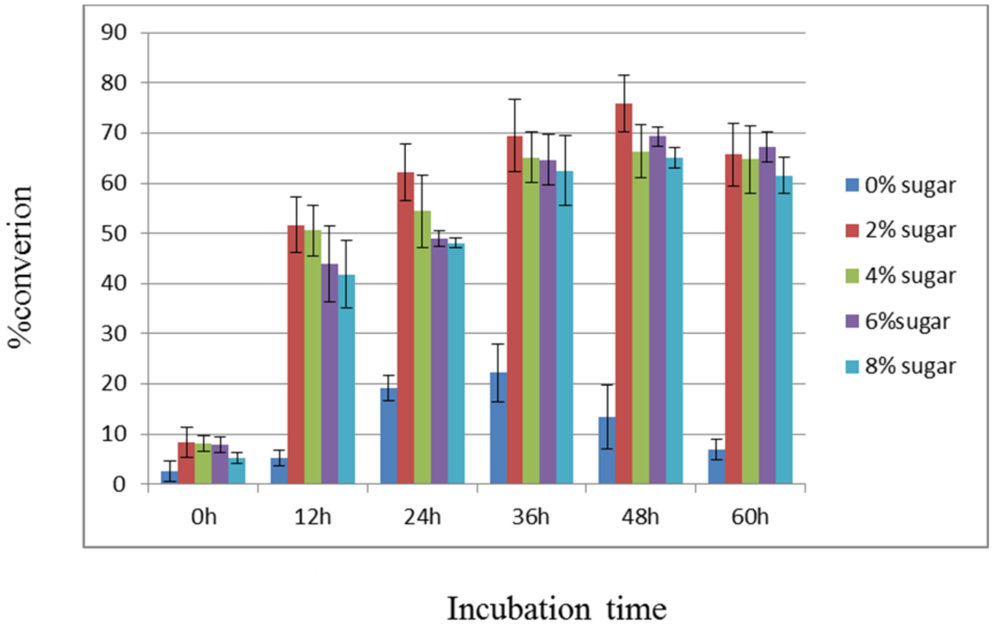

Fig. S4. HPLC chromatogram of various concentration sugar adding at 48h with quinirazin as substrate. A. 0%; B. 2%; C.4%; D.6%; E.8%. S refers to the substrate peak and P refers to the product.

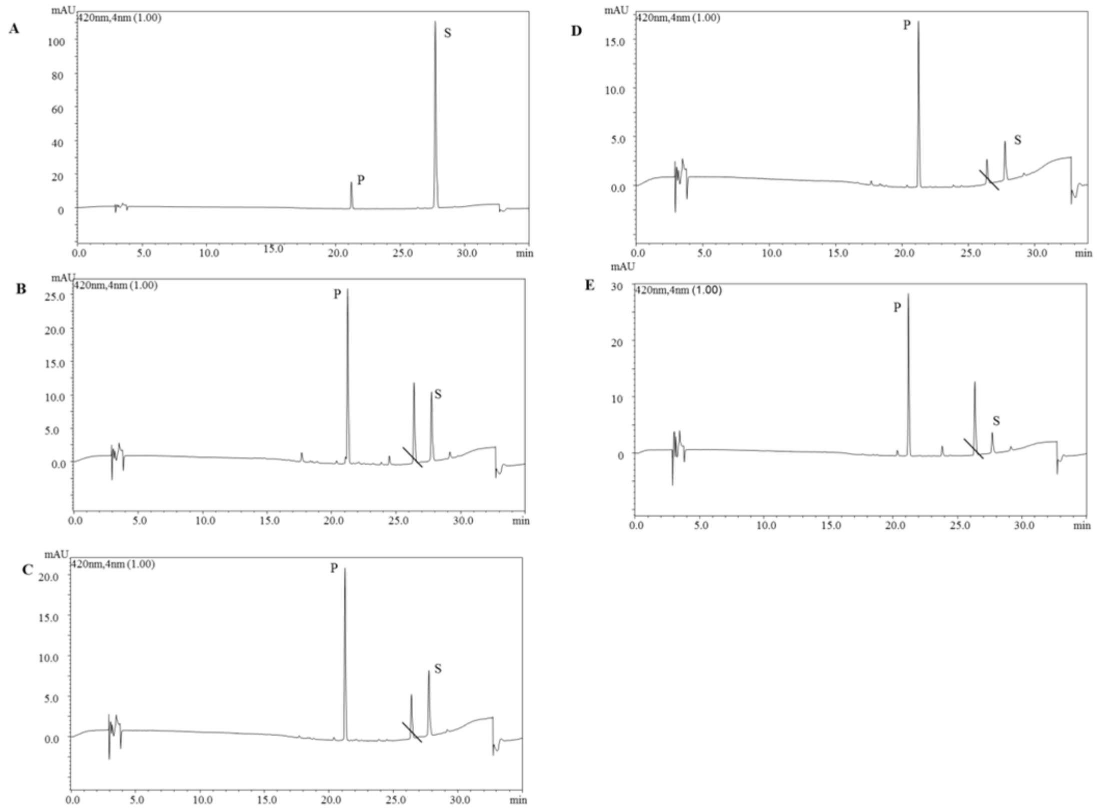

Fig. S5. (a) ^{1}H- NMR of quizarin

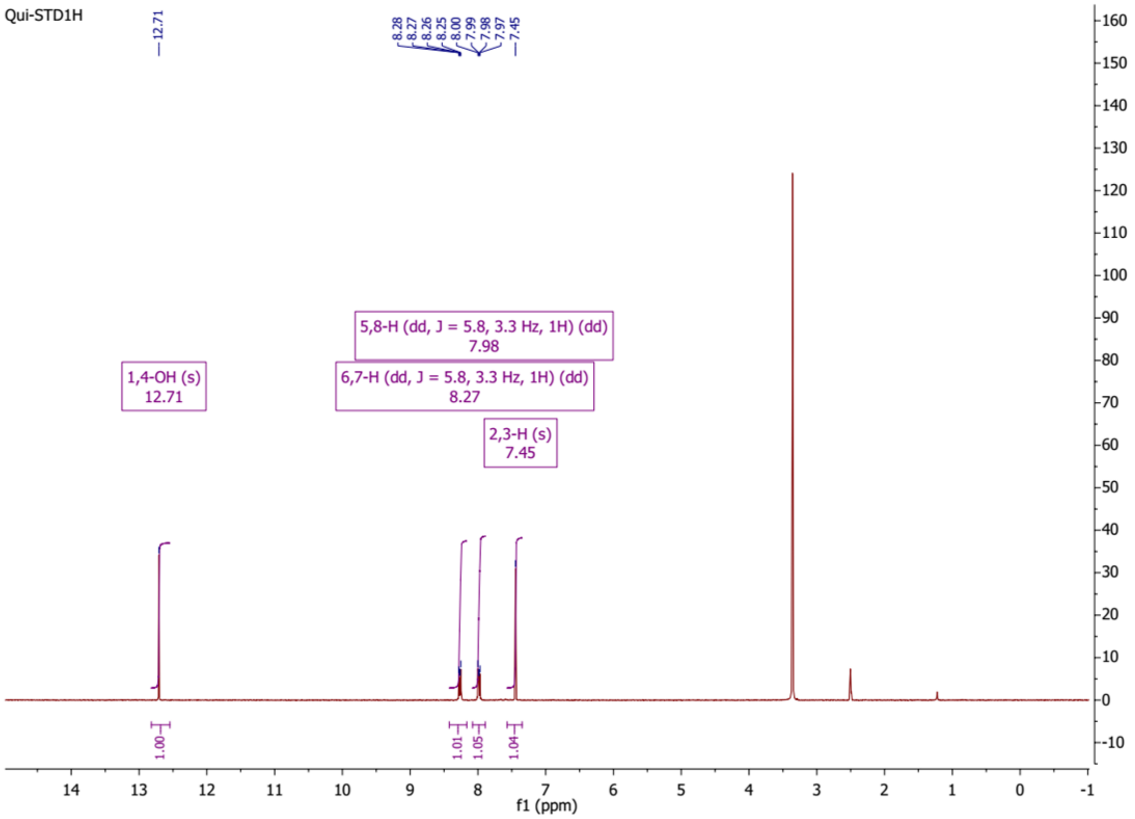

Fig. S5. (b) ^{1}H- NMR quinizarin -4-*O*-*α*- rhamnoside

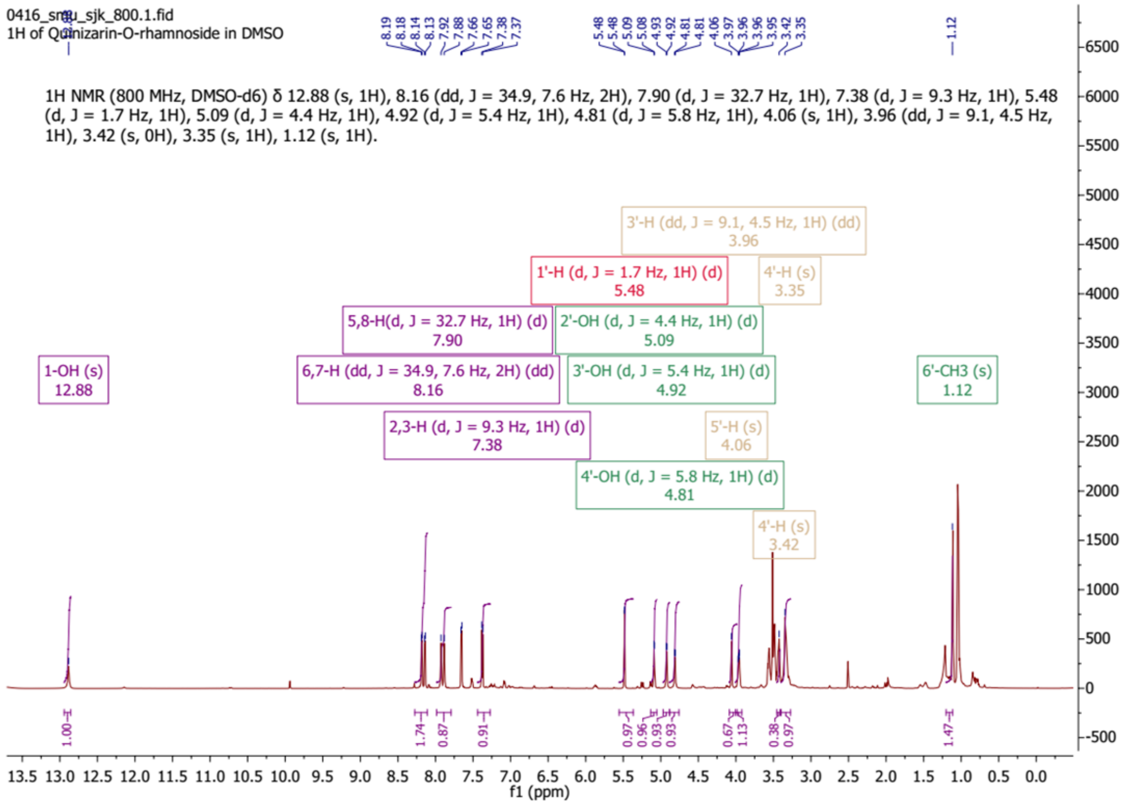

Fig. S6. (a) ^{13}C- NMR of quizarin

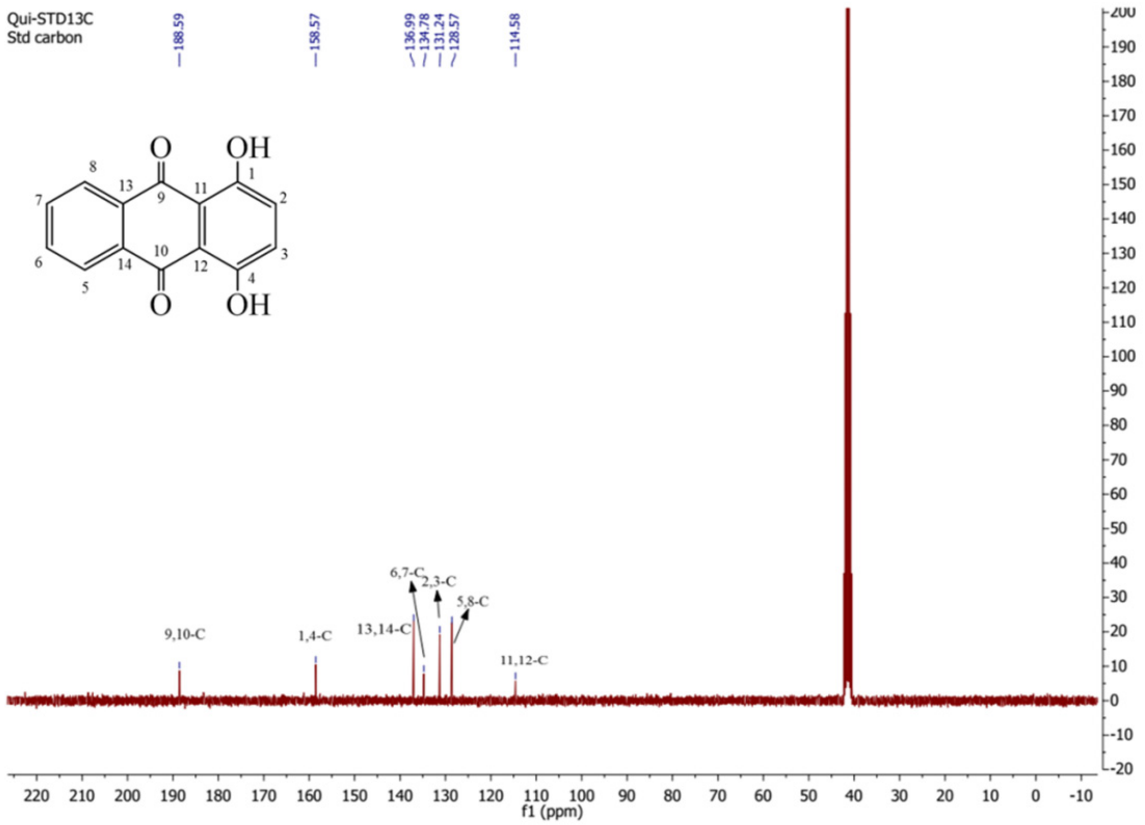

Fig S6 (b) ^{13}C- NMR quinizarin -4-*O*-*α*- rhamnoside

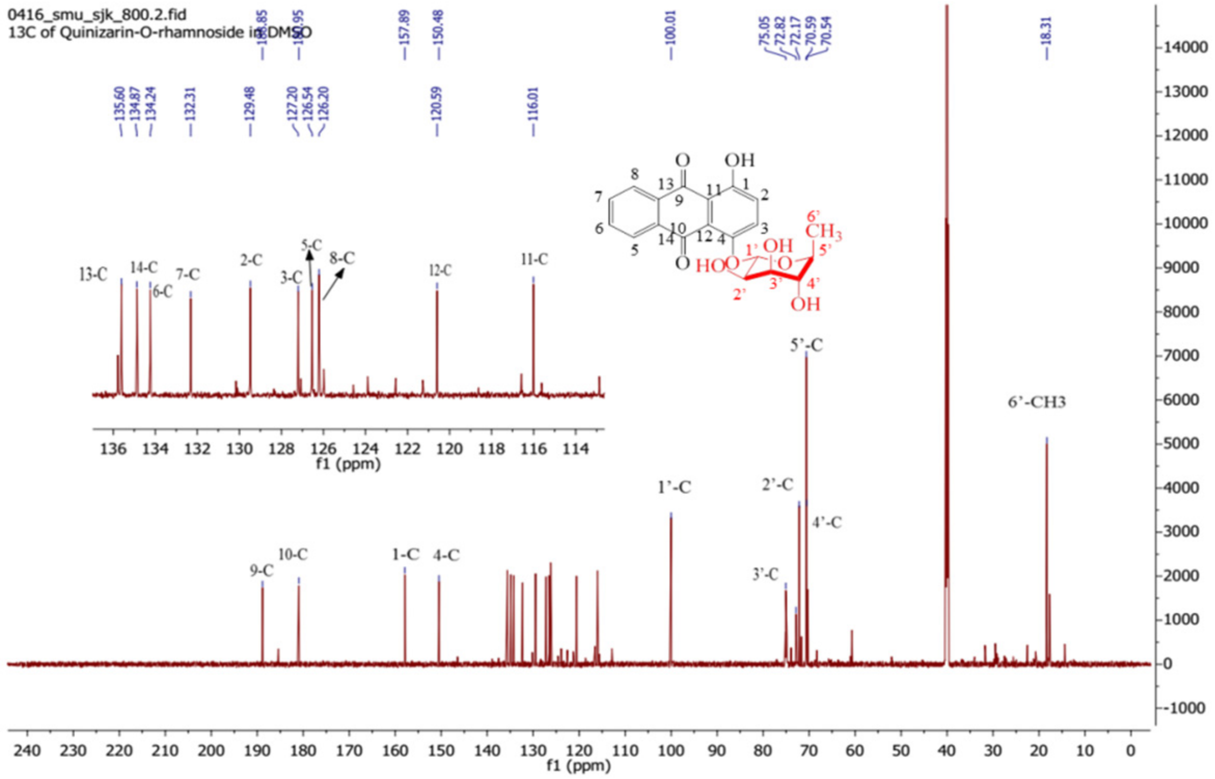

Fig. S7. HSQC correlation of quinizarin -4-*O*-*α*- rhamnoside

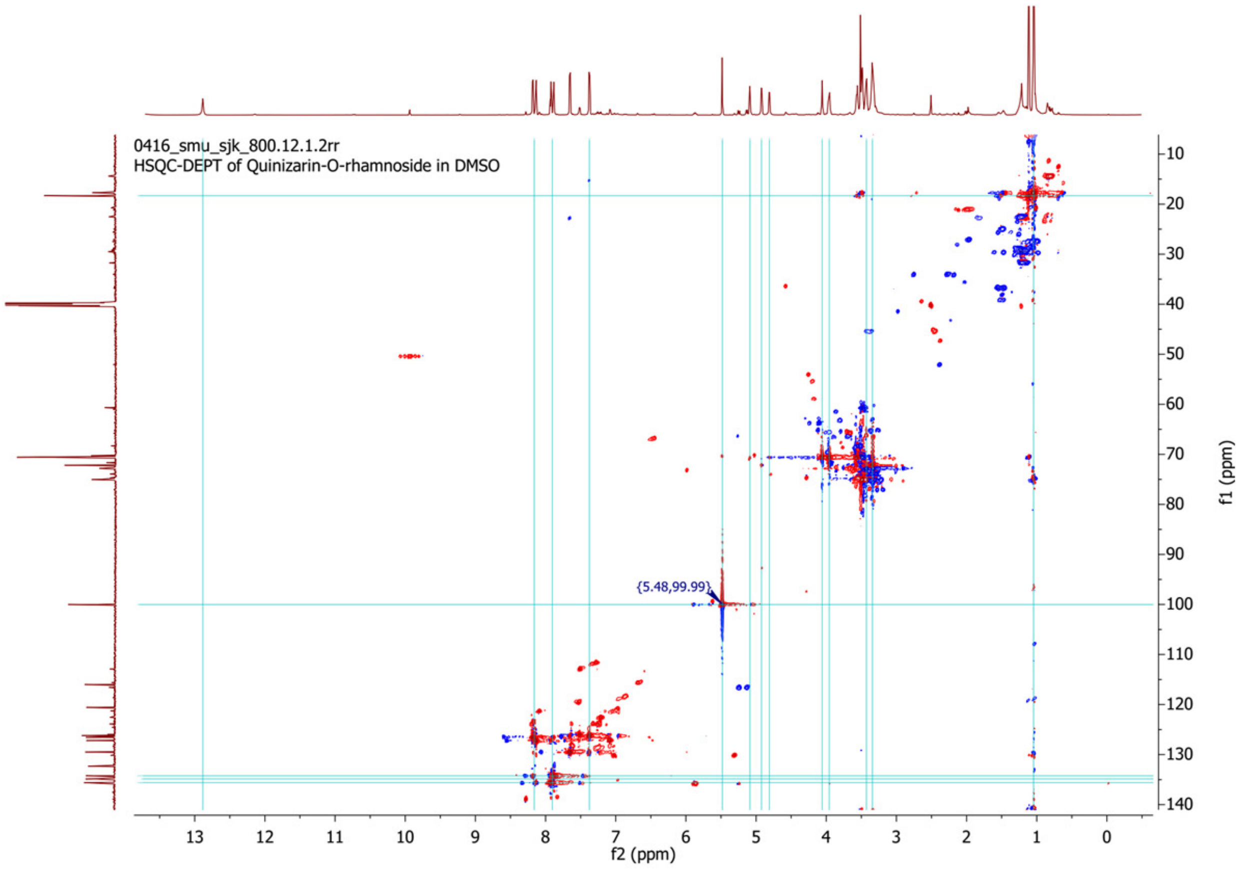

Fig. S8. HMBC correlation of quinizarin -4-*O*-*α*- rhamnoside

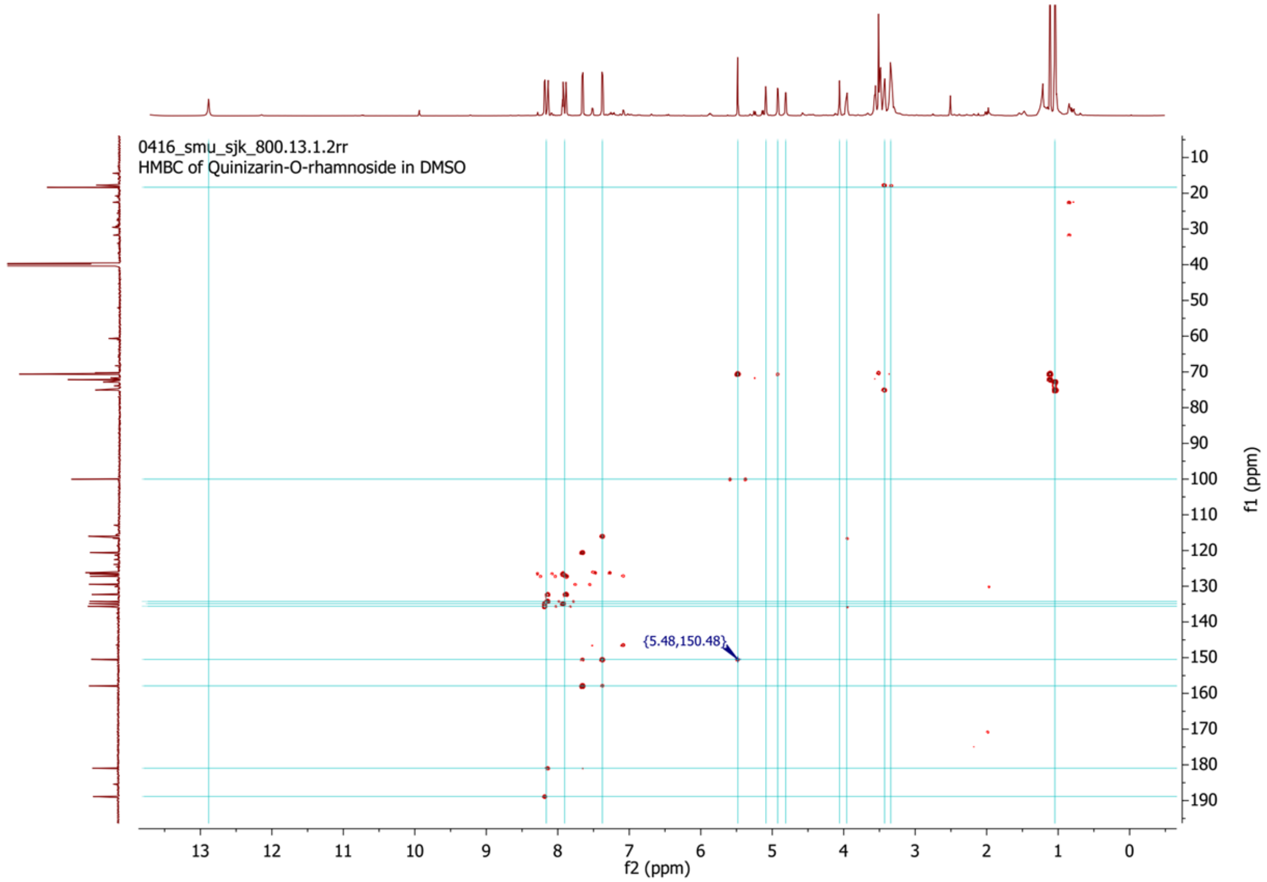

Reference

1. Datsenko KA, Wanner BL. 2000. One-step inactivation of chromosomal genes in Escherichia coli K-12 using PCR products. *Proc. Natl. Acad. Sci. USA* **97**: 6640–6645.
2. Pandey RP, Parajuli P, Chu LL, Darsandhari S, Sohng JK. 2015. Biosynthesis of amino deoxy-sugar-conjugated flavonol glycosides by engineered *Escherichia coli. Biochem. Eng. J.* **101**: 191–199.
3. Strobel T, Schmidt Y, Linnenbrink V, Luzhetskyy A, Luzhetska M, Taguchi T, *et al.* 2013. Tracking down biotransformation to the genetic level: Identification of a highly flexible glycosyltransferase from *Saccharothrix espanaensis*. *Appl. Environ. Microbiol.* **79**: 5224 –5232 .
4. Nguyen THT, Pandey RP, Parajuli P, Han JM, Jung HJ, Park YI, *et al.* 2018. Microbial Synthesis of Non-Natural Anthraquinone glucosides display in superior antiproliferative properties. *Molecules* **23**: 2171
5. Heo JR , Kim SM, Hwang KA , Kang JH, Choi KC. 2018. Resveratrol induced reactive oxygen species and endoplasmic reticulum stress-mediated apoptosis, and cell cycle arrest in the A375SM malignant melanoma cell line. *Int. J. Mol. Med*. **42**: 1427-1435.
6. Aakvaag A, Utaaker E, Thorsen T, Lea OA, Lahooti H. 1990. Growth control of human mammary cancer cells (MCF-7 Cells) in culture: Effect of estradiol and growth factors in serum-containing medium. *Cancer Res*. **50**: 7806-7810.
7. Li N, Zhang P, Kiang KMY, Cheng YS, Leung GKK. 2018. Caffeine sensitizes U87-MG human glioblastoma cells to temozolomide through mitotic catastrophe by impeding G2 arrest. *BioMed. Res. Int*. **2018**: 1–10.
